# A Role for PGC-1a in the Control of Abnormal Mitochondrial Dynamics in Alzheimer’s Disease

**DOI:** 10.3390/cells11182849

**Published:** 2022-09-13

**Authors:** Jia Wang, Wen-Jun Liu, Hou-Zhen Shi, Hong-Ru Zhai, Jin-Jun Qian, Wei-Ning Zhang

**Affiliations:** 1 The Fourth Affiliated Hospital of Jiangsu University, Zhenjiang 212001, China; 2 Department of Laboratory Medicine, School of Medicine, Jiangsu University, Zhenjiang 212013, China

**Keywords:** Alzheimer’s disease, PGC-1a, mitochondrial distribution, mitochondrial dynamics, mitochondrial dysfunction

## Abstract

Emerging evidence suggests that the proper control of mitochondrial dynamics provides a window for therapeutic intervention for Alzheimer’s disease (AD) progression. The transcriptional coactivator peroxisome proliferator activated receptor gamma coactivator 1 (PGC-1a) has been shown to regulate mitochondrial biogenesis in neurons. Thus far, the roles of PGC-1a in Alzheimer’s disease and its potential value for restoring mitochondrial dysfunction remain largely unknown. In the present study, we explored the impacts of PGC-1a on AD pathology and neurobehavioral dysfunction and its potential mechanisms with a particular focus on mitochondrial dynamics. Paralleling AD-related pathological deposits, neuronal apoptosis, abnormal mitochondrial dynamics and lowered membrane potential, a remarkable reduction in the expression of PGC-1a was shown in the cortex of APP/PS1 mice at 6 months of age. By infusing AAV-*Ppargc1α* into the lateral parietal association (LPtA) cortex of the APP/PS1 brain, we found that PGC-1a ameliorated AD-like behavioral abnormalities, such as deficits in spatial reference memory, working memory and sensorimotor gating. Notably, overexpressed PGC-1a in LPtA rescued mitochondrial swelling and damage in neurons, likely through correcting the altered balance in mitochondrial fission–fusion and its abnormal distribution. Our findings support the notion that abnormal mitochondrial dynamics is likely an important mechanism that leading to mitochondrial dysfunction and AD-related pathological and cognitive impairments, and they indicate the potential value of PGC-1a for restoring mitochondrial dynamics as an innovative therapeutic target for AD.

## 1. Introduction

Alzheimer’s disease (AD), characterized by neurofibrillary tangles, amyloid-β (Aβ) aggregates and progressive neuronal loss in the brain, is the most prevalent neurodegenerative disease in the aged population [1]. Although the culprit of AD pathology remains elusive, accumulating evidence shows the involvement of altered mitochondrial dynamics and distribution in the onset and progression of synaptic and neuronal degeneration [1]. Neurons, or nerve cells, are highly polarized cells composed of distinct structural regions: the soma, dendrites and a long axon [2]. The elaborate structure of the neuron requires a regulatory mechanism for distributing a sufficient number of mitochondria to its axon for energy supply, calcium homeostasis, neuronal plasticity, synaptic transmission and apoptosis [3]. Existing in both sporadic and familial forms, the AD brain is characterized by signs of axonal degeneration as noticed by defects in mitochondrial trafficking and distribution [2,4]. Specifically, using transmission electron microscopy, the region-specific depletion of synaptic mitochondria was observed in the brains of AD patients [5]. Consistently, utilizing a drosophila AD model, Iijima-Ando et al. [6] confirmed the association of AD mechanisms with mitochondrial mislocalization; specifically, Aβ42 reduced the number of mitochondria in axons with the concomitant accumulation of mitochondria in the soma. The extensive regionalized neuronal loss in AD brains is marked by damage to axonal integrity and an alteration in the distribution pattern of mitochondria that precedes neuronal death [7]. Interestingly, it was noticed that the reduction in the mitochondria in axons was followed by an increase in mitochondria size, suggesting that mitochondrial trafficking and mitochondrial fusion–fission are interlinked [1,2].

Increasing evidence has revealed that mitochondrial distribution is orchestrated by fission/fusion processes to meet specific subcellular energetic supply and calcium buffering needs [7]. The dynamic machinery involves large dynamin-related GTPases that exert opposing effects, e.g., optic atrophy protein 1 (OPA1) and mitofusins (MFN1 and MFN2) for fusion and dynamin-related protein 1 (DRP1) and fission protein 1 (Fis1) for fission with the assistance of other factors in neurons [7]. Emerging evidence suggests that mitochondrial function is regulated by the dynamics of its membrane fusion–fission; longer, fused mitochondria are optimal for ATP generation, whereas the fission of mitochondria facilitates mitophagy and cell division [7].

Although the molecular mechanism underlying the pattern of neurodegeneration remains murky, accumulating evidence shows that abnormalities of mitochondrial fusion–fission seem to take center stage in AD pathology [8]. Studies of APP-overexpressed cells confirmed that mutant APP caused imbalanced mitochondrial fission–fusion that resulted in mitochondrial fragmentation [1]. Notably, the GSK-3β-mediated phosphorylation of *Drp1* at the Ser693 residue induces elongated mitochondrial morphology that in turn inhibits mitochondrial fission and transport from the soma to the distal axons [8]. Altogether, these studies illustrated that targeting AD-specific mitochondrial fusion–fission and trafficking can provide a window for developing novel therapeutics for AD. Drugs or peptides that can increase synaptic function and ameliorate axonal integrity in neurons represent an innovative therapeutic strategy for AD.

As a transcriptional coactivator coordinates, peroxisome proliferator activated receptor PPARγ coactivator-α (PGC-1a) is strongly expressed in the heart, skeletal muscle, liver, kidney, brown adipose tissue and brain [9]. The purported roles of PGC-1a include, but are not limited to, (1) promoting mitochondrial biogenesis and oxidative phosphorylation [10]; (2) promoting the expression of several reactive oxygen species (ROS) detoxifying enzymes and preventing oxidative stress by inhibiting the production of ROS. However, the role of PGC-1a and its involvement in AD is largely unknown. Based on study findings that PGC-1a inhibits Rotenone-induced dopaminergic neurotoxicity involved in the regulation of both fusion and fission proteins, thus determining mitochondrial network structure [11], and that PGC-1a stimulates the activity of the MFN2 promoter [12], which mediated mitochondrial transport and the expression of motor protein in human spinal motor neurons [13], we contend that PGC-1a potentially plays an important role in regulating mitochondrial dynamics and function in neurons in AD. Thus, this study can be divided into two parts as follows: (1) to investigate the association of *Pgc-1alpha* gene variants with AD-relevant neuronal apoptosis and the protein expression involved in mitochondrial fusion and fission, as well as mitochondrial distribution, morphology and membrane potential (MMP); and (2) to explore the intervention effects of PGC-1a on AD pathology and AD-like neurobehavioral abnormalities and its potential mechanism with a particular focus on mitochondrial dynamic.

## 2. Materials and Methods

### 2.1. Patients

We obtained 4 brain specimens of patients older than 70 years with a clinical diagnosis of AD and 3 controls from the Chinese Brain Bank Center (CBBC). Controls were defined as non-demented subjects according to clinical records. Subjects were excluded if they had suffered from a neurological disease other than AD, as revealed by clinical, neuroradiological or neuropathological evaluation. All subjects were Chinese and completed the Mini Mental State Examination (MMSE) and dementia-rating scale (DRS) evaluation (Table 1). The investigations were carried out following the rules of the Declaration of Helsinki of 1975 (https://www.wma.net/what-we-do/medical-ethics/declaration-of-helsinki, accessed on 8 September 2022), revised in 2013, and the protocol was approved by the Ethics Committee of Jiangsu University (UJS-IACUC-AP-2022022525).

### 2.2. Animals

All of the animals were housed in an animal vivarium under a reversed light–dark cycle (lights on 1900~0700 h) and kept under ad libitum food and water conditions throughout the entire study. Following AAV infusion, the animals were caged singly and handled daily. All behavioral manipulations were conducted in the dark phase of the cycle. Experiments protocols were approved (1) by the Committee on Humane Use of Animals at Jiangsu University (SYXK2018-0053) and (2) by the National Institutes of Health Guide for the Care and Use of Laboratory Animals (NIH Publications No. 8023, revised 1978).

### 2.3. APP/PS1 Mice

*APP/PS1* double transgenic (2×Tg-AD) mice were obtained from Jackson Laboratory (No. 34829-JAX), and the genotypes of the offspring were determined as previously reported [14]. In 2×Tg-AD animals; Aβ-plaques were first obviously observed at 6 months of age and increased over time [14]. As such, 6-month-old male mice were chosen for all experiments.

### 2.4. PGC-1a Overexpressed in the Cortex of AD Mice

APP/PS1 mice were anesthetized with xylazine/ketamine (11.6/73 mg/kg, i.p) and placed in a stereotaxic frame (RWD Life Science) with a mouse adapter. The tip of a pulled glass pipette was inserted at stereotaxic coordinates for AP −1.94 mm, for ML- ±1.5 mm and for DV- −1.0 mm, relative to the bregma, according to our previously report [10,15]. Viral Vector suspension in a volume of 0.5 μL of PBS buffer was microinjected into the bilateral lateral parietal association (LPtA) cortex of APP/PS1 genotype mice using 2 μL bursts from a Nanoliter 2000 injector (World Precision Instruments). This corresponded to 2 × 10^12^ viral genome copies (vgc) of pAAV-MCS-*Ppargc1α*-m-FLAG-HA and 1 × 10^12^ vgc of pAAV-MCS-FLAG-control; expression plasmid was provided by Applied Biological Material co (abm, Zhenjiang, China). It was reported that AAV-mediated transgenic protein expression peaked after three weeks and remains at stable levels after infusion [16,17]. Accordingly, all behavioral tests and the subsequent molecular examinations were performed three weeks later after mice were infused with AAV. Virus expression was corroborated by immunofluorescence and western blot.

### 2.5. Experimental Design

In Experiment 1, two genotypes including WT (*n* = 8) and 2×Tg-AD (*n* = 8) were applied to confirm the associations of *Pgc-1alpha* gene variants with AD pathology and abnormal mitochondrial dynamics. In Experiment 2, two treatments including AAV-Vector (*n* = 8) and AAV-*Pgc-1alpha* (*n* = 8) were infused into the LPtA cortex of APP/PS1 mice to explore the role of PGC-1a in AD as well as its potential mechanisms in rescuing the disturbance of mitochondrial dynamics.

### 2.6. Cell Lines, Plasmids and Transfection

Unless stated otherwise, all culture media were purchased from Invitrogen (Carlsbad, CA, USA), and reagents and chemicals were purchased from Sigma-Aldrich (St. Louis, MO, USA). N_2_A neuroblastoma cells were purchased from the Shanghai Academy of Sciences (cat # TCM29, Shanghai, China). These cells were transfected at ~40% confluency using Lipofectamine 2000 reagent (cat # SCSP-502, Life Technologies, Shanghai, China). For the co-transfections of two plasmids, the cells were transfected at a ratio of 2 µg: 2 µg. The control plasmids including *pEnCMV* and *pCDNA* 3.0 as well as the expression plasmids including pCAX *APPswe*/Ind and *pEnCMV*-*Pargc1a*-Flag were purchased from Miaoling Bio (Wuhan, China).

### 2.7. Western Blot

N_2_A cells were transfected with the Vector plasmid or plasmid-encoding *APPswe* or co-transfectioned with *APPswe* and *pEnCMV*/*Ppargc1a* plasmids for 48 h, cell homogenate was lysed and Western blots were performed as described previously [14]. Primary antibodies against EGFP (1:1000, Beyotime, cat # AG281, Shanghai, China), Flag (1:1000, abm, cat # G188, Zhenjiang, China), OPA1 (1:1000, Boster, cat # PB0773, Wuhan, China), MFN1 (1:1000, Boster, cat # PB0263, Wuhan, China), MFN2 (1:800, Bioss, cat # bs-23685R, Beijing, China), DRP1 (1:1000, Wanleibio, cat # WL03028, Shenyang, China), FIS1 (1:1000, Boster, cat # A01932-2, Wuhan, China), BAX (1:5000, Abcam, cat # ab32503, Cambridge, MA, USA), Bcl-2 (1:2000, Abcam, cat # ab182858, Cambridge, MA, USA), PGC-1a (1:1000, Bioss, cat # bs-1832R, Beijing, China), KIF5A Flag (diluted 1:1000, Bioword, cat # BS71526, Nanjing, China), KIF5B (diluted 1:1000, Wanleibio, cat # WL04906, Shenyang, China), and the horseradish peroxidase-linked antibodies (1:5000, Beyotime, goat anti mouse IgG, cat # A0216 and goat anti rabbit IgG, cat # A0208, Shanghai, China) were used to probe these blots. Protein visualization was carried out using enhanced chemiluminescence (ECL, Beyotime Institute of Biotechnology, Shanghai, China). The signal intensity was obtained by densitometric scanning.

### 2.8. Immunofluorescence

Mice were anesthetized and perfused, and their brains were fixed. We prepared 6-μm-thick sections from the parietal cortex. To visualize PGC-1a expression and Aβ deposits, sections were first incubated with primary antibodies, including goat anti-PGC-1a (1:200, Bioss, cat # bs-1832R, Beijing, China), mouse anti-Aβ (1:200, CST, cat # D3D2N, Boston, MA, USA), mouse anti-GFP (1:200, Beyotime, cat # AF2882, Shanghai, China), mouse anti-Flag (1:200, Beyotime, cat # AF2852, Shanghai, China) and mouse anti-HA (1:200, Bioss, cat # bsm-33003M, Beijing, China) overnight at 4 °C. After being rinsed in PBT (0.5% Triton X-100 in 1 × PBS), sections were incubated for 2 h at 25 °C with FITC goat anti-mouse IgG (H + L) (1:500, Beyotime, cat # A0568, Shanghai, China) and Alexa Fluor 594 goat anti-rabbit IgG (H + L) (1:1000, CST, cat # 8889, Boston, MA, USA). After several rinses in PBT, the sections were mounted using antifade mounting medium (Beyotime, cat # P0126, Shanghai, China), and fluorescence was visualized using a fluorescence microscope (BX41, Olympus, Tokyo, Japan).

### 2.9. Immunohistochemistry

After anesthesia and perfusion, the brains of the mice were fixed. Coronal frozen sections (6 μm) from the parietal cortex were used to investigate the parietal cortices of the brains. Brain sections were immunolabeled with an antibody against OPA1 (1:200, cat # PB0773, Boster, Wuhan, China), MFN2 (1:100, Bioss, cat # bs-23685R, Beijing, China), DRP1 (1:200, Wanleibio, cat # WL03028, Shenyang, China), FIS1 (1:200, Boster, cat # A01932-2, Wuhan, China) and incubated with the corresponding secondary antibody. After washing, DAB (Boster, cat # AR1022, Wuhan, China) was used for dyeing, and hematoxylin was used for retaining. The sections were dehydrated in a graded series of ethanol and xylene and mounted using non-aqueous mounting medium. Selected images were captured using a SPOT camera and software system (Diagnostic Instruments, Sterling Heights, MI, USA) attached to a Leica DM5000B microscope (Leica, Bannockburn, IL, USA) with 4× objectives.

### 2.10. Apoptosis Assay

The Annexin V-FITC apoptosis detection kit (Vazyme, cat # A211-01, Nanjing, China) was used to assess apoptosis. After transfecting, N_2_A cells were trypsinized and collected by centrifugation at 1000 rpm for 5 min, washed with cold PBS and resuspended in 0.5 mL of buffer containing 5 μL of Annexin V and 5 μL of propidium iodide. The samples were incubated at 37 °C for 15 min in the dark and analyzed using flow cytometry within 1 h. The CytExpert instrument (Beckman Coulter) was used to determine the apoptotic rate.

### 2.11. Scanning Electron Microscopy

A series of 50 μm thick sections 250 μm apart from each other were collected from the parietal cortices of the animals. After washing in PBS, sections were treated with 0.5% osmium-tetroxide for 20 min, dehydrated and embedded in epoxy resins. During dehydration, sections were treated with 1% uranyl acetate. After polymerization, we prepared 70 nm thick sections (Leica EM UC7) from the parietal cortex, picked them up on formvar-coated single-slot copper grids and examined them using a JEOL-1200EX electron microscope (EM) and a Soft Imaging System Veleta CCD camera (EMSIS, Münster, Germany). For measurements of images of electron microscopy, we used Image J software.

### 2.12. Probes for Mitochondria

N_2_A cells were grew on cover slips in 6-well plates at a density of 70–90% and were transfected with corresponding plasmids for 48 h. Medium was then replaced with prewarmed media containing MitoTracker Deep Red FM (Sigma-Aldrich, Merck KGaA, Darmstadt, Germany) at 400 nM. Dishes were incubated in the dark at 37 °C for 30 min, and then fixed in 4% paraformaldehyde for 10 min at room temperature. After washing with PBS, cells were permeabilized with 0.3% Triton X-100 for 15 min. and then incubated with blocking solution containing 1% BSA for 1 h. When the blocking was accomplished, cells were incubated with corresponding primary antibody, secondary antibody and DAPI. For each sample, confocal images were collected in fluorescence modes, followed by electronic merging of the images using a confocal microscope (MRC1024ES; BioRad, Hercules, CA, USA).

### 2.13. Nissl Staining

Brain sections were embedded in paraffin. The tissue sections (6 µm) were cleared using xylene and rehydrated with anhydrous ethanol at 95, 80 and 70% alcohol and double-distilled water. Sections were then stained in 0.1% cresyl-violet acetate (Solarbio, Beijing, China) at 56 °C for 1h. The tissues were rinsed in double-distilled water and differentiated in 70% ethanol with acetic acid for 1 min. Subsequently, dehydrated with 70, 80 and 95% alcohol, washed in xylene and placed on a cover slip with slide mounting medium. Neuronal cell counting in the parietal cortex was conducted using a light microscope. Image-Pro Plus 6.0 (Media Cybernetics, Inc., Rockville, MA, USA) software was used to count the number of dead neurons.

### 2.14. Flow Cytometry Detection of JC-1 Fluorescence

The changes in mitochondrial MMP were measured with an MMP assay kit with JC-1 (Solarbio, cat # M8650, Beijing, China) according to the manufacturer’s instructions. Briefly, treated N_2_A cells were harvested and washed with cold PBS. Then, the cells were suspended in a mixture of 1 mL of culture medium and 1 mL of JC-1 staining working solution (10 μg/mL) for 20 min in the dark at 37 °C. Subsequently, the cells were washed with cold staining buffer three times. Afterwards, cells were counted in a BD Accuri C6 flow cytometer (BD Bioscience, San Jose, CA, USA).

### 2.15. Fluorescence of JC-1 Stained Treated Cells

Similarly, cells suspended in a mixture of 1 mL of culture medium and 1 mL of JC-1 staining solution (10 μg/mL) were also used for fluorescence microscope (BX41, Olympus, Tokyo, Japan). Samples were evaluated using 488 nm excitation and the registration of both green and red fluorescence. Samples were evaluated using NIS-Elements software (Nikon CEE GmbH, Vienna, Austria).

### 2.16. Imaging and Analyses

For each animal, digital images from 6-8 brain sections were acquired in a fluorescence microscope and the number of cells positive for a specific marker was counted using image J software.

### 2.17. Behavioral Test Battery and Data Collection

Tests were conducted one per day and were ordered from least to more intrusive to minimize the effects of each test on the following ones [15]. The order of the tests was novel object recognition (NOR), prepulse inhibition of acoustic startle reflex (PPI) and Morris water maze (MWM) test. The procedures of the behavioral tests followed our previously reported methods [15]. The entire behavioral pattern of the animals during the experimental trials was recorded with a video camera and a video monitor located in an adjacent room. The measurements and data analyses were performed using Ethovision 8.5 software (Noldus, Wageningen, The Netherlands). The Startle Reflex software system (SR-LAB, San Diego Instruments, San Diego, CA, USA) was used to assess PPI.

### 2.18. Statistical Analyses

Unpaired Student’s *t*-test was used for imaging, electron microscopic analysis and immunoblotting data (Graph Pad Prism version 6.0, La Jolla, CA, USA). Behavioral data from PPI and MWM tests were subjected to repeated ANOVA. Post hoc comparisons were conducted using Fisher’s protected least significant difference test. Significant differences were accepted at *p* < 0.05. All values are presented as means ± S.E.M.

## 3. Results

### 3.1. A Significant Reduction in PGC-1a Expression Is Observed in AD

**Figure 1:**
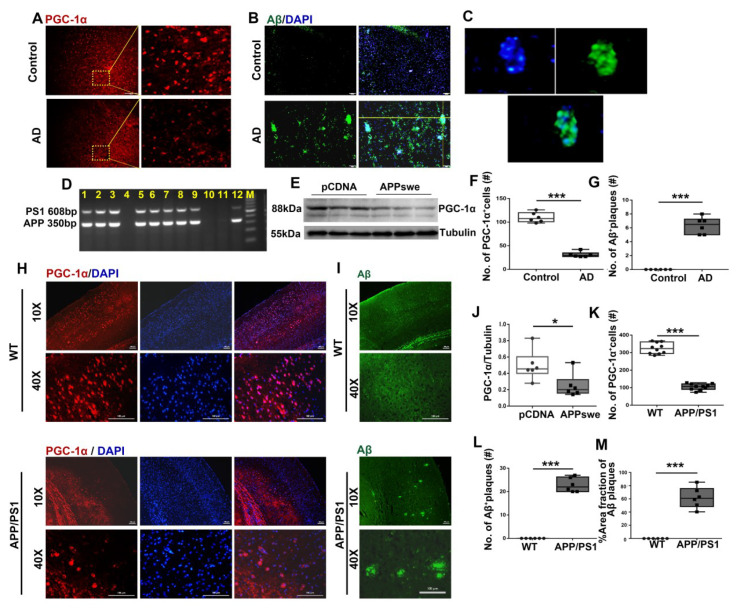
A remarkable reduction in PGC-1a expression is observed in AD patients, cells and 2×Tg-AD mice. Expression patterns and qualification of (**A**,**F**) PGC-1a and (**B**,**C**,**G**) Aβ deposits from the parietal cortex samples of AD and control (Ctr) humans were analyzed using immunofluorescence. N_2_A cells were transfected with *pCDNA* plasmid or plasmid-encoding APP (*APPswe*) for 48 h. (**E**) Expression patterns and (**J**) qualification of PGC-1a were studied with Western blot. (**D**) PCR products obtained from genomic DNA from APP/PS1 mice. The 608 and 350 bp bands resulted from the amplification of PS1 and APP alleles, respectively. Expression patterns and qualification of (**H**,**K**) PGC-1a and (**I**,**L**,**M**) Aβ deposits from the parietal cortex samples of APP/PS1 and WT mice (6 months) were studied with immunofluorescence. The qualification of % Area fraction of Aβ plaques was calculated using the formula [area fraction of Aβ plaques/total area fractions] × 100% between the two genotypes. Scale bars = 200 μm. For each group, *n* = 6–10. Significance levels were set at * *p* < 0.05, *** *p* < 0.001 for noted differences between Ctr and AD groups, *pCDNA* and *APPswe* groups or WT and APP/PS1 groups. Tubulin was used as the loading control.

To investigate the association of the PGC-1a varies with the occurrence of AD, we first investigated cortical specimens from AD patients. Using Aβ and PGC-1a antibodies, we found that increased aggregation in Aβ plaques (Figure 1B,C,G) was accompanied by reduced expression in PGC-1a levels (Figure 1A,F) in the cortex of AD patients than the control ones, suggesting the relevance of PGC-1a varies with AD pathology.

To expand the present results, we expressed *APPswe* plasmid in N_2_A cells. A remarkable reduction in PGC-1a expression was observed in the *APPswe*-transfected cells compared with the control (Ctr) ones (Figure 1E,J).

Utilizing APP/PS1, a 2×Tg-AD mouse line (Figure 1D), we investigated the association of AD-relevant pathological deposition with PGC-1a expression. As shown in Figure 1I, a remarkable increase in Aβ aggregates was found in APP/PS1 mice at 6 months of age (Figure 1L,M). Accordingly, all experiments were conducted in 6-month-old mice in this study. Again, using antibody against PGC-1a, we observed that reduced PGC-1a expression was exhibited in the cortices of 2×Tg-AD mice compared with that in wild-type (WT) mice (Figure 1H,K).

### 3.2. Regulation Effect of MutAPP on Mitochondrial Fission and Fusion Proteins

**Figure 2:**
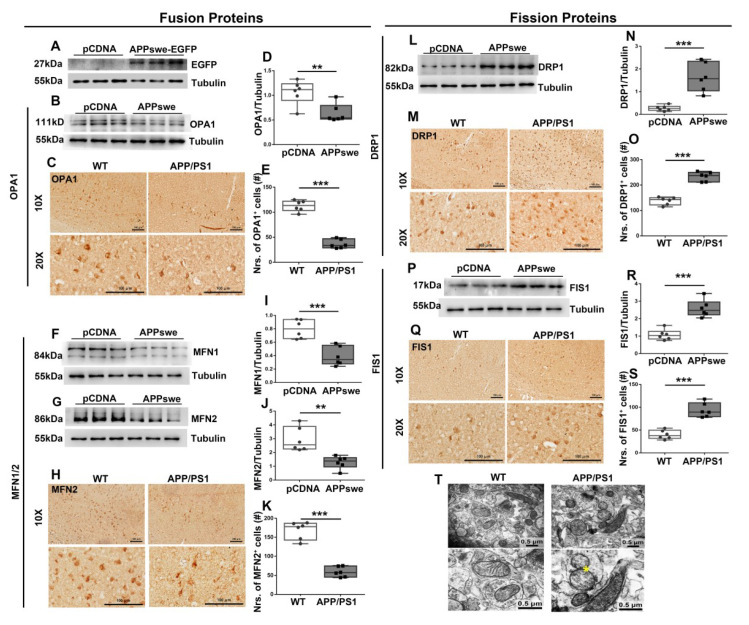
MutAPP triggers dynamic imbalance of mitochondrial fission and fusion. N_2_A cells were transfected with *pCDNA* or *APPswe* plasmid for 48 h. (**A**) EGFP-labeled *APPswe* was successfully overexpressed in N_2_A cells. Expression patterns and qualification of the fusion proteins, including (**B**,**D**) OPA1, (**F**,**I**) MFN1 and (**G**,**J**) MFN2, as well as the fission proteins, including (**L**,**N**) DRP1 and (**P**,**R**) FIS1 were studied with Western blot. Expression patterns and qualification of (**C**,**E**) OPA1, (**H**,**K**) MFN2, (**M**,**O**) DRP1 and (**Q**,**S**) FIS1 from the parietal cortex samples of 2×Tg-AD mice were also examined with immunohischemistry. Scale bars = 100 μm. (**T**) Representative electron microscope images showing stages of mitochondria in cortical neurons of WT and APP/PS1 animals. Scale bar = 0.5 μm. Yellow stars: the fissive stage of mitochondria. Significance levels were set at ** *p* < 0.01, *** *p* < 0.001 for noted differences between *pCDNA* and *APPswe* groups and WT and APP/PS1 groups. Tubulin was used as the loading control.

We next investigated the effect of mutAPP on the expression of mitochondrial fusion (i.e., OPA1, MFN1, MFN2) and fission proteins (i.e., DRP1, FIS1). N_2_A cells were transfected with Vector or *APPswe* plasmid for 48 h, (A) EGFP-labeled *APPswe* was successfully overexpressed in N_2_A cells. Immunoblot analysis revealed that OPA1 (Figure 2B,D), MFN1 (Figure 2F,I) and MFN2 (Figure 2G,J) was significantly reduced whereas DRP1 (Figure 2L,N) and FIS1 (Figure 2P,R) remarkably increased in *APPswe*-transfected cells compared with Ctr ones.

To extend the present results, we further examined the cortical expression of fusion (i.e., OPA1 and MFN2) and fission proteins (i.e., DRP1 and FIS1) in 2×Tg-AD mice using immunohischemistry analysis. Consistently, decreased OPA1 (Figure 2C,E) and MFN2 expression (Figure 2H,K) and increased DRP1 (Figure 2M,O) and FIS1 levels (Figure 2Q,S) were observed in the cortices of 2×Tg-AD animals than the Ctr ones.

In addition, using transmission electron microscopy (TEM) technology, we observed that more mitochondria were in a fissive stage from cortical neurons of AD brain (Figure 2T).

### 3.3. MutAPP Induces a Remarkable Increase in Neuronal Apoptosis

**Figure 3:**
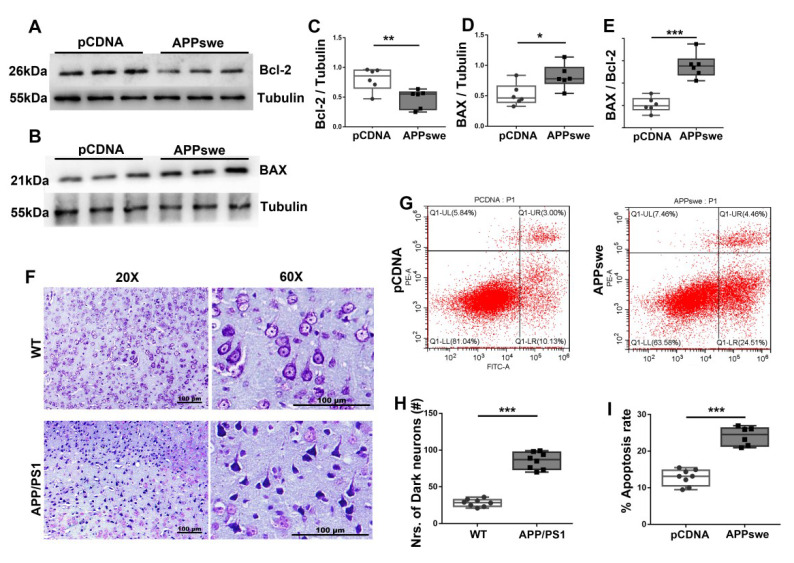
Increased neuronal apoptosis is observed in AD cells and 2×Tg-AD animals. N_2_A cells were transfected with *pCDNA* or *APPswe* plasmid for 48 h. Expression patterns and qualification of (**A**,**C**) B cell lymphoma/leukemia-2 (Bcl-2) and of (**B**,**D**) Bcl-2 associated X protein (BAX) in the cells were studied with Western blot (**E**) Apoptosis index, defined as the expression ratio of BAX to Bcl-2, is illustrated with the bar graph. (**F**) Representative images and (**H**) qualification of Nissl staining for the parietal cortex samples from APP/PS1 and WT mice are illustrated. Scale bar = 100 μm. (**G**,**I**) N_2_A cells were transfected with *pCDNA* or *APPswe* plasmids for 48 h, and the % apoptotic rate was examined by CytExpert flow cytometry. For each group, *n* = 6–8. Significance levels were set at * *p* < 0.05, ** *p* < 0.01, *** *p* < 0.001 for noted differences between *pCDNA* and *APPswe* groups and WT and APP/PS1 groups, Tubulin was used as the loading control.

To confirm the notion that apoptosis contributes to AD pathology [18], using N_2_A cell line, we compared the expression of B cell lymphoma/leukemia-2 (Bcl-2) and Bcl-2 associated X protein (BAX), two parameters that are associated with cell apoptosis between *pCDNA*- and *APPswe*-transfected cells. A remarkable increase in neuronal apoptosis was revealed by the changes of (Figure 3A,C) Bcl-2 and (Figure 3B,D) BAX expression and (Figure 3E) the apoptosis index [BAX/Bcl-2], as well as by the apoptosis rate (Figure 3G,I).

Using Nissl staining technology, we further confirmed that mice transgenic for APP and PS1 also influence neuronal apoptosis. In the WT group, neuron cells with normal size and morphology, clear nucleus and Nissl substance in the cytoplasm were tightly packed and orderly arranged, while neurons with loss of Nissl substance and abnormal nuclear shape were irregularly arranged in the cortices of 2×Tg-AD mice (Figure 3F). There were significantly darker neurons in the parietal cortices of APP/PS1 mice than in those of WT mice (Figure 3H).

### 3.4. Effects of MutAPP Expression on Mitochondrial Axonal Transport and Distribution

**Figure 4:**
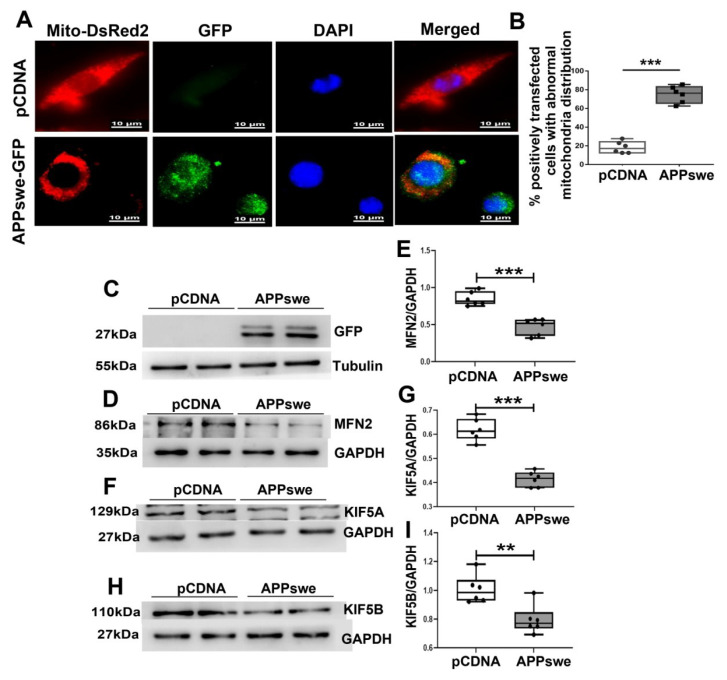
MutAPP causes abnormal mitochondrial distribution. (**A**) Representative confocal pictures of mitochondria in N_2_A cells transected with *pCDNA* or GFP-tagged *APPswe* plasmid. Cells were incubated with Mito-DsReds to label mitochondria. Red = Mito-DsRed2, Green = GFP-labeled *APPswe*, Blue = DAPI. Scale bars = 10 µm. (**B**) The qualification of % positively transfected cells with abnormal mitochondria distribution was calculated using the formula [Numbers of cells with abnormal mitochondria distribution /total positively transfected cells] ×100% between the two groups. At least 20 cells were analyzed in triplicate for each cell line. *n* = 6 for each group. Significance levels were set at *** *p* < 0.001 for noted differences between *pCDNA* and *APPswe*-transfected cells. (**C**) N_2_A cells were transfected with *pCDNA* or *APPswe* plasmid for 48 h. GFP-labeled *APPswe* was successfully overexpressed in N_2_A cells. Expression patterns and qualification of (**D**,**E**) MFN2, (**F**,**G**) KIF5A and of (**H**,**I**) KIF5B in the cells were studied with Western blot. For each group, *n* = 6. Significance levels were set at ** *p* < 0.01, *** *p* < 0.001 for noted differences between *pCDNA* and *APPswe* groups, GAPDH was used as the loading control.

A widely used mitochondrial marker, Mito-DsReds, was used to trace mitochondrial distribution in cells. To confirm that the abnormal distribution of mitochondria contributes to AD pathology, we transfected N_2_A cells with *pCDNA* or GFP-tagged *APPswe* plasmid. In line with a previous report [19], mitochondrial distribution in the positively *APPswe* -transfected cells is perinuclear, with few mitochondria in the distal axons where they are normally distributed in healthy Ctr cells (Figure 4A). Notably, the percentage of cells with mitochondrial clustering around the perinuclear area increased significantly from 18.36 ± 2.58% in APPswe-transfected cells to 75 ± 3.67% in Ctr ones (Figure 4B).

MFN2, a key molecule regulated by PGC-1a [12], has been shown to facilitate anterograde axonal mitochondrial transport [13]. Therefore, the expression of MFN2 was studied by western blot. As showed in Figure 4C, D and E, a remarkable reduction in MFN2 expression was induced by transfecting with *APPswe* plasmids. As a member of the kinesin-1 family, KIF5b is responsible for mitochondrial axonal transport [20]. The three isoforms of KIF5b include Kinesin Family Member 5A (KIF5A), Kinesin Family Member 5B (KIF5B), and Kinesin Family Member 5C (KIF5C). KIF5B is ubiquitous, while KIF5A and C are neuronal [20]. Because any problem in the attachment of KIF5 and mitochondria will rapidly impair mitochondrial transport and lower mitochondrial count in neurons’ end terminals [21], the variants of KIF5A and KIF5B were examined in the present study. As expected, mutAPP expression caused a remarkable reduction in KIF5A and KIF5B expression (Figure 4F–I). 

### 3.5. Effect of MutAPP Expression on Mitochondrial Morphology and Membrane Potential

**Figure 5:**
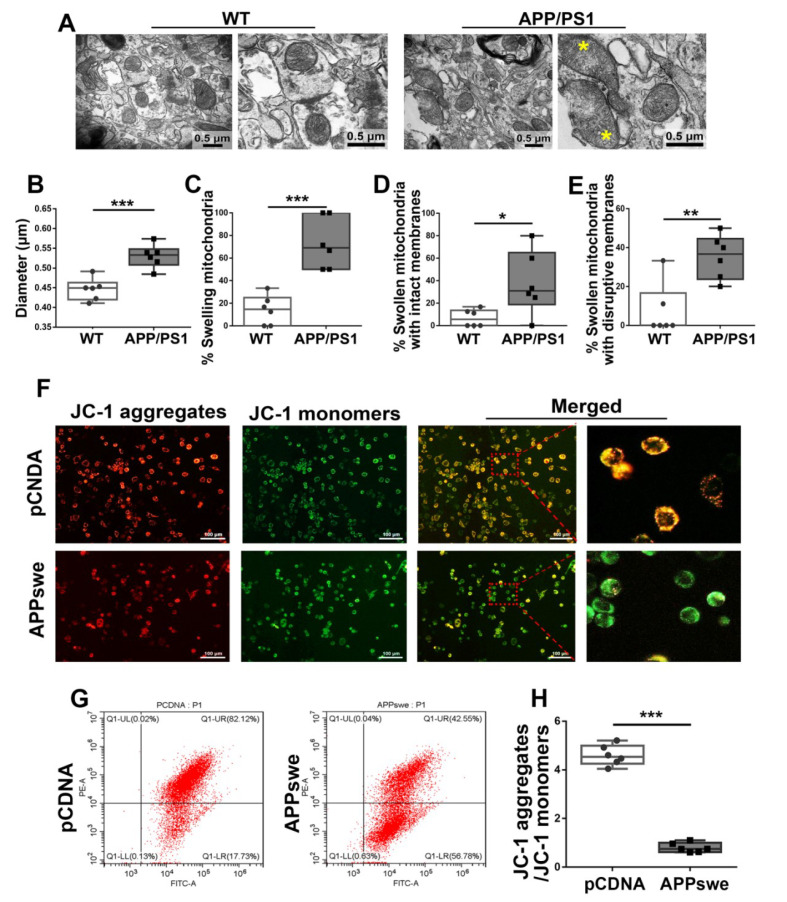
Abnormal mitochondrial morphology and lowered MMP are showed in AD. (**A**) Electron micrograph showing representative mitochondrial morphology from parietal cortex samples of WT and APP/PS1 transgenic animals. Yellow star: swollen mitochondria. Scale bar = 0.5 μm. Quantitative analysis showing the (**B**) diameters of mitochondria and (**C**–**E**) proportions of swelling mitochondria both with intact membranes and with ruptured ones in cortical neurons of WT and APP/PS1 brains. For each group, *n* = 6/group. Significance levels were set at * *p* < 0.05, ** *p* < 0.01, ***p < 0.001 for noted differences between WT and APP/PS1 groups. N_2_A cells were transfected with *pCDNA* plasmid or plasmid-encoding *APPswe* for 48 h. Detection of JC-1 signals in N_2_A cells was performed using fluorescence confocal microscopy. Data are representative of three independent measurements. (**F**) Fluorescence was registered using excitation at 488 nm and adjusting the emission of confocal microscopy for J-monomers (visible as green) and J-aggregates (visible as red/orange). (**G**,**H**) The ratios of J-aggregates to J-monomers as an indicator of mitochondrial membrane potential (MMP) were examined with CytExpert flow cytometry. For each group, *n* = 6/group. Significance levels were set at *** *p* < 0.001 noted difference between *pCDNA* and *APPswe* groups.

Axonal transport (mitochondrial transport or axonal transport of mitochondria) is necessary for removing dysfunctional and/or damaged mitochondria [22]. Therefore, it was suggested that impaired axonal transport should be linked to changes in mitochondrial morphology and function. The morphology of the mitochondria in the cortical area of each group was determined using TEM (Figure 5A). The mean diameter of the mitochondria from WT brains was 0.45 μm, while mice transgenic for APP and PS1 induced an increase (0.53 μm) in the mean diameter of cortical mitochondria (Figure 5B). Notably, more round and enlarged mitochondria were shown in the cortex of the APP/PS1 brain (Figure 5C) and swelling mitochondria either with intact membranes (Figure 5D) or with disrupted membranes (Figure 5E).

It has been shown that the disturbance of mitochondrial distribution and morphology is unable to meet the metabolic demands of the cells and as a result inevitably affects synaptic function [23]. Therefore, we used fluorescence microscopy to detect J-aggregates and J-monomers in *pCDNA*/*APPswe*-transfected cells after their incubation with JC-1. In control cells with higher MMP, JC-1 spontaneously forms J-aggregates that emit red fluorescence in the mitochondria. However, in *APPswe*-transfected cells, the MMP declines, and JC-1 is released from the mitochondria and exists as a monomer with green fluorescence in the cytoplasm (Figure 5F).

To extend the present study, we also monitored MMP by flow cytometry analysis of JC-1 staining (Figure 5G). The mean fluorescence intensity of the JC-1 aggregates of the control cells was 78.81%, and in the respective dot plots, they were positioned in the upper right quadrant in Figure 5G,H. In contrast, *APPswe*-transfected cells demonstrated JC-1 aggregates with a mean fluorescence intensity of 42.86%. MutAPP significantly reduced MMP as indicated by the reduced percentages of aggregates and monomers (Figure 5H).

### 3.6. PGC-1α Is Forced Expressed in the LPtA Cortex of APP/PS1 Brains

**Figure 6:**
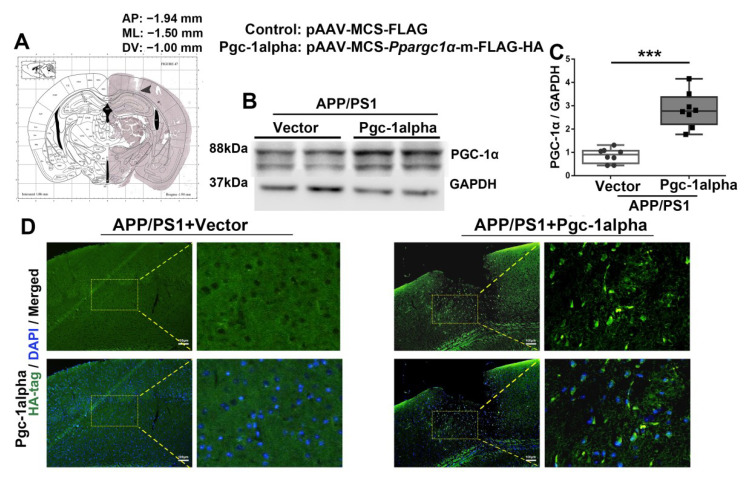
rAAV2/9 forces PGC-1α overexpressed in the LPtA cortex of APP/PS1 mice. Photomicrograph of cresyl violet stained coronal section from the brain of a mouse with representative placement in the bilateral lateral parietal association cortex (LPtA). (**A**) Location of infusion sites in the LPtA of genotype mice. (**B**,**C**) Cortical lysates of the two treatment mice were immunoblotted using an antibody against PGC-1α. Values are expressed as means ± S.E.M. For each group, *n* = 8/group. Significance levels were set at *** *p* < 0.001 for noted differences between AAV-Vector- and AAV-*Pgc*-*1alpha*-infused AD animals. (**D**) Representative immunofluroscence images showed AAV2/9 forced PGC-1α overexpression in the LPtA cortex of APP/PS1 mice. Green = HA−labeled PGC-1α; Blue = DAPI. Scale bars = 100 μm.

To gain more insight into the role of PGC-1α in mitochondrial dynamics and neurobehavioral abnormalities in AD, the Vgc of pAAV-MCS-*Ppargc1α*-m-FLAG-HA (AAV-*Pgc-1alpha*) was microinjected into LPtA cortices of APP/PS1 brains to force PGC-1α overexpression (Figure 6A). The immunoblot analysis of two treatments using an antibody specific for PGC-1α revealed that the parietal cortex in AAV-*Pgc-1alpha* infusion mice exhibited a remarkable increase in the expression of PGC-1α as compared with AAV-Vector infusion ones (Figure 6B,C). Immunofluorescence using the HA antibody double confirmed that AAV-*Pgc-1alpha* infusion induced a global increase in PGC-1α expression in the parietal cortex of 2×Tg-AD animals (Figure 6D).

### 3.7. Effect of PGC-1α Expression on Dynamic Imbalance of Mitochondrial Fission and Fusion in AD

**Figure 7:**
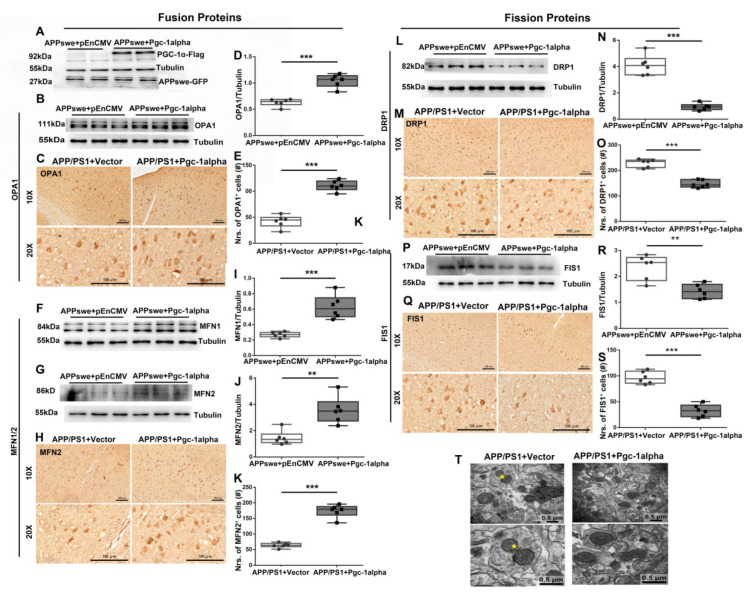
PGC-1α rescues mutAPP-triggered dynamic imbalance of mitochondrial fission and fusion. N_2_A cells were transfected with *pEnCMV*/*Pgc-1alpha* plasmid and plasmid-encoding *APPswe* for 48 h. (**A**) Flag-labeled PGC-1α and EGFP-labeled *APPswe* were successfully overexpressed in N_2_A cells. Expression patterns and qualification of the fusion proteins, including (**B**,**D**) OPA1, (**F**,**I**) MFN1 and (**G**,**J**) MFN2, as well as the fission proteins, including (**L**,**N**) DRP1 and (**P**,**R**) FIS1, were studied with Western blot. Expression patterns and qualification of (**C**,**E**) OPA1, (**H**,**K**) MFN2, (**M**,**O**) DRP1 and (**Q**,**S**) FIS1 from the parietal cortex samples of 2×Tg-AD mice treated with Vector/*Pgc-1alpha* were also examined with immunohistochemistry. Scale bars = 100 μm. (**T**) Representative electron microscopes showing stage of mitochondria in cortical neurons of AAV-Vector- and AAV-*Pgc-1alpha* treated APP/PS1 mice. Scale bar = 0.5 μm. Yellow stars: the fissive stage of mitochondria. Significance levels were set at ** *p* < 0.01, *** *p* < 0.001 for noted differences between *APPswe* + *pEnCMV* and *APPswe* + *Pgc-1alpha* groups, or APP/PS1 + Vector and APP/PS1 + *Pgc-1alpha* groups. Tubulin was used as the loading control.

To test the intervention effect of PGC-1α on the variants of mitochondrial fusion/fission proteins in AD, N_2_A cells were co-transfected with *APPswe* and *pEnCMV*/*Pgc-1alpha* plasmid for 48 h, and GFP-labeled *APPswe* and Flag-labeled PGC-1α were examined to confirm the success of transfection (Figure 7A). Immunoblot analysis revealed that PGC-1α significantly increased OPA1 (Figure 7B,D), MFN1 (Figure 7F,I) and MFN2 (Figure 7G,J) and significantly reduced DRP1 (Figure 7L,N) and FIS1 (Figure 7P,R) expression in *APPswe*-transfected cells.

Further, utilizing APP/PS1 mouse line combined with AAV-*Pgc-1alpha* infusion, we observed that PGC-1α contributes to ameliorating abnormal mitochondrial dynamics, as indicated by promoting the expression of mitochondrial fusion proteins including OPA1 (Figure 7C,E) and MFN2 (Figure 7H,K) and inhibiting the levels of mitochondrial fission proteins, such as DRP1(Figure 7M,O) and FIS1(Figure 7Q,S).

Additionally, using TEM technology, an obvious inhibition in mitochondrial fission was observed in the cortical neurons of AAV-*Pgc-1alpha*-infused AD animals (Figure 7T).

### 3.8. Effect of PGC-1α Expression on Neuronal Apoptosis in AD

**Figure 8:**
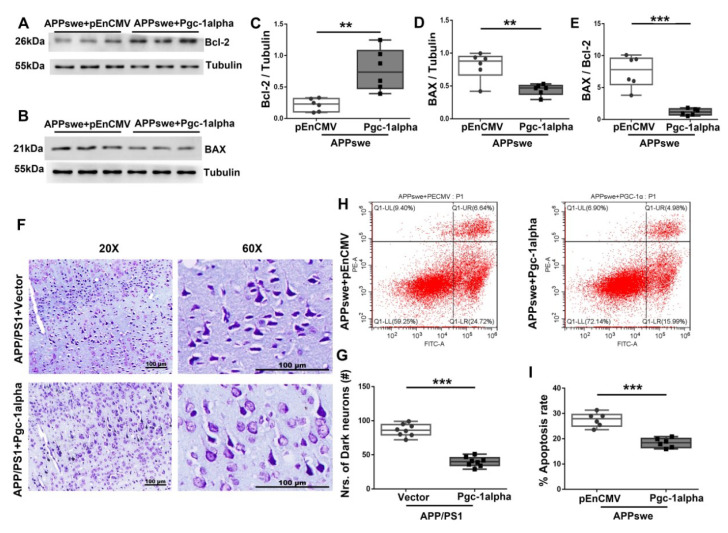
PGC-1α ameliorates apoptosis-relevant AD pathology. Expression patterns and qualification of (**A**,**C**) Bcl-2 and (**B**,**D**) BAX from the cortical samples of AAV-*Pgc-1alpha*-treated AD brains are showed. (**E**) Anti-apoptotic index, defined as the expression ratio of Bcl-2 to BAX was illustrated by bar graph. (**F**) Representative images of Nissl staining from the parietal cortex samples of Vector- and *Pgc-1alpha-* treated AD mice. Scale bar = 100 μm. (**G**) Nrs of dark neurons of Nissl substance between the two treatments were qualified and showed by bar graph. (**H**) Apoptosis rate was analyzed with flow cytometry after overexpressing *pEnCMV*/*Pgc-1alpha* and plasmid-encoding *APPswe* for 48 h in N_2_A cells. (**I**) Quantification of proportion of apoptotic cells of the two treatments were displayed by bar graphs. Values are expressed as means ± S.E.M. For each group, *n* = 6–8/group. Significance levels were set at ** *p* < 0.01, *** *p* < 0.001 for noted differences between *APPswe* + *pEnCMV* and *APPswe* + *Pgc-1alpha* groups, or APP/PS1 + Vector and APP/PS1 + *Pgc-1alpha* groups. Tubulin was used as the loading control.

To determine the role of PGC-1α in apoptosis-relevant AD pathology, we examined the expressions of Bcl-2 and BAX, two parameters that are associated with cell apoptosis. As shown in Figure 8A–D, PGC-1α decreased BAX but increased Bcl-2 expression in *APPswe*-transfected cells. Again, quantitative analysis confirmed the anti-apoptotic role of PGC-1α, as revealed by a remarkable reduction in apoptosis index [BAX/Bcl-2] (Figure 8E).

To extend the present study findings, we investigated neuronal morphology using Nissl staining technology and found that irregular morphologies with loss of Nissl substance were exhibited in the neurons from the cortex of the APP/PS1 brain; however, by infusing AAV-*Pgc-1alpha*, an obvious inhibition in neuronal apoptosis was displayed in the cytoplasm of AD cortices. Specifically, an integrative and granular-like morphology characterized as clear nucleus and tightly packed Nissl substance is shown in Figure 8F,G.

Thereafter, using flow cytometry technology, we analyzed the effect of PGC-1α expression on the changes in *APPswe*-induced cell apoptosis. As shown in Figure 8H,I, PGC-1α expression significantly inhibited mutAPP-induced neuronal apoptosis.

### 3.9. Effect of PGC-1α Expression on Mitochondrial Axonal Transport and Distribution in AD

**Figure 9:**
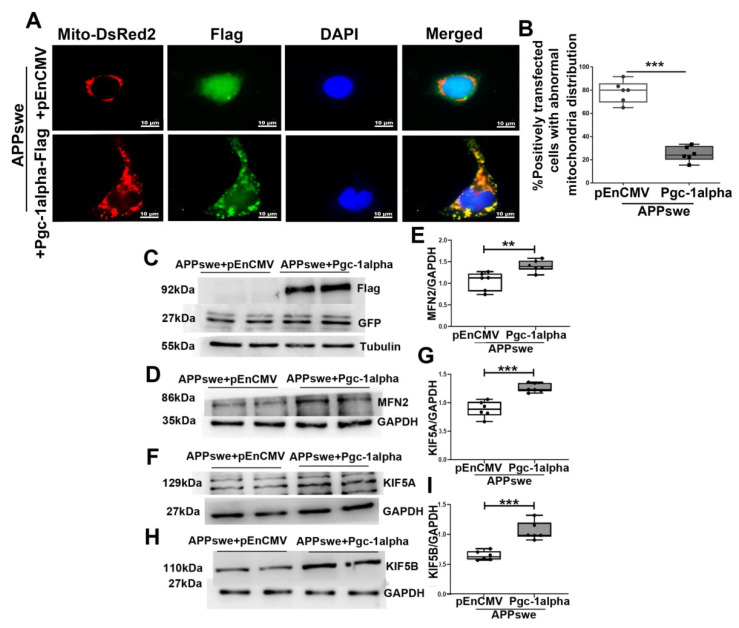
PGC-1α antagonizes mutAPP-induced abnormalities in mitochondrial axonal transport and distribution. (**A**) Representative confocal pictures of mitochondria in N_2_A cells transected with *APPswe* and *pEnCMV*/*Pgc-1alpha* plasmids. Cells were incubated with Mito-DsReds to label mitochondria. Red = mito-DsRed2, Green = Flag-labeled PGC-1α, Blue = DAPI. Scale bars = 10 µm. (**B**) The qualification of % positively transfected cells with abnormal mitochondria distribution was calculated using the formula [Numbers of cells with abnormal mitochondria distribution /total positively transfected cells] ×100% between the two treatments. At least 20 cells were analyzed in triplicate for each cell line. *n* = 6 for each group. Significance levels were set at *** *p* < 0.001 for noted differences between *APPswe* + *pEnCMV* and *APPswe* + *Pgc-1alpha* transfected cells. (**C**) N_2_A cells were transfected with *pEnCMV*/*Pgc-1alpha* plasmid and plasmid-encoding *APPswe* for 48 h. GFP-labeled *APPswe* and Flag-labeled *Pgc-1alpha* were successfully overexpressed in N_2_A cells. Expression patterns and qualification of (**D**,**E**) MFN2, (**F**,**G**) KIF5A and of (**H**,**I**) KIF5B in the cells were studied with Western blot. For each group, *n* = 6. Significance levels were set at ** *p* < 0.01, *** *p* < 0.001 for noted differences between *APPswe* + *pEnCMV* and *APPswe* + *Pgc-1alpha* groups. Tubulin/GAPDH was used as the loading control.

Because mutAPP changes mitochondrial morphology and induces mitochondrial malfunction, we sought to determine whether PGC-1α can ameliorate dynamical abnormalities of mitochondria. N_2_A cells were transfected with *pEnCMV*/*Pgc-1alpha* and *APPswe* edited plasmids; the distribution of mitochondria was labeled using Mito-DsRed (Figure 9A).

In *APPswe*-transfected cells, 65% to 91.7% of cells demonstrated an abnormal distribution with mitochondria accumulating around the perinuclear area, whereas more remote cytoplasmic areas and axons were devoid of mitochondria. However, by transfection with *Pgc-1alpha* plasmids, cells were observed in which mitochondria were distributed evenly throughout the cytoplasm and axon (>66.7%) (Figure 9B). 

Motor-based anterograde mitochondrial transport along axons is primarily driven by kinesins; therefore, we examined the regulated effect of PGC-1α on expression of MFN2 and KI5A/B, the transport-associated motor proteins in AD cells (Figure 9C). It is of interest to note that PGC-1α overexpression promoted the expression of MFN2 (Figure 9D,E), KIF5A (Figure 9F,G) and B (Figure 9H,I), which are responsible for mitochondrial transport, adaptor binding and axonal maintenance [24].

### 3.10. Effect of PGC-1α Expression on Mitochondrial Morphology and Membrane Potential in AD

**Figure 10:**
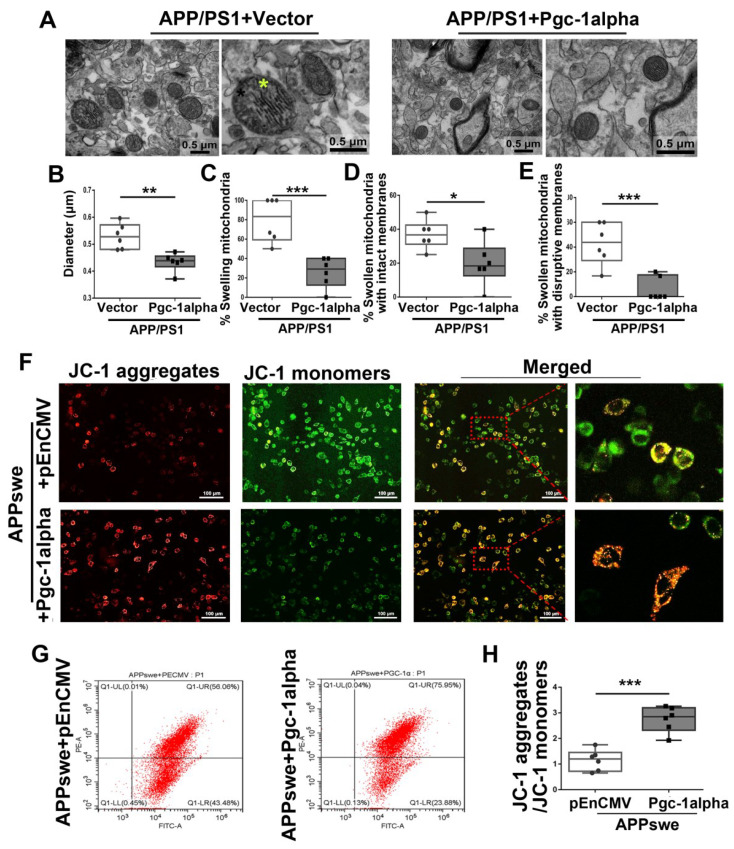
PGC-1α rescues AD-associated morphological abnormalities and MMP declines. (**A**) Representative electron microscopies showing mitochondrial morphology in axons in vivo. Yellow star: swollen mitochondria. Scale bar = 0.5 μm. Quantitative analysis showing the treatment effect of AAV-Vector or AAV-*Pgc-1alpha* on (**B**) diameter of mitochondria and (**C**–**E**) number of swelling mitochondria both with intact membranes and with ruptured ones in AD brains. For each group, *n* = 6/group. Significance levels were set at * *p* < 0.05, ** *p* < 0.01, *** *p* < 0.001 for noted differences between APP/PS1 + Vector and APP/PS1 + *Pgc-1alpha* groups. (**F**) N_2_A cells were transfected with *pEnCMV*/*Pgc-1alpha* and plasmid-encoding *APPswe* for 48 h. Detection of JC-1 signals in N_2_A cells was performed with fluorescence confocal microscopy. Data are representative of three independent measurements. Fluorescence was registered using excitation at 488 nm and adjusting the emission of confocal microscopy for J-monomers (visible as green) and J-aggregates (visible as red/orange). (**G**,**H**) The ratio of J-aggregates to J-monomers as an indicator of mitochondrial MMP was examined with CytExpert flow cytometry. For each group, *n* = 6. Significance levels were set at *** *p* < 0.001 for noted differences between *APPswe* + *pEnCMV* and *APPswe* + *Pgc-1alpha* groups.

As to mitochondrial morphology, most cells in the cortices of APP/PS1 animals had a swollen mitochondrial structure. Specifically, the mean diameter of mitochondria in the cortex of APP/PS1 mice was 0.53 μm, while *Pgc-1alpha* infusion induced a significant decrease (0.43 μm) in the mean diameter of the cortical mitochondria (Figure 10A,B) that even returned to the baseline level (WT group) (Figure 5A). We also examined the numbers of swelling mitochondria; notably, *Pgc-1alpha* infusion reduced the percentages of swelling mitochondria with both intact and disrupted membranes (Figure 10C–E).

To further explore the effect of PGC-1α activation on mitochondrial function, N_2_A cells were transfected with *APPswe* and *pEnCMV*/*Pgc-1alpha* plasmids. Confocal microscopy was used to evaluate mitochondrial MMP by observing J-aggregates and J-monomers in cells after their incubation with JC-1 probes. In *APPswe* and *pEnCMV* co-transfected cells, lowered MMP was displayed as green fluorescence for the J-monomer and weaker red fluorescence for the J-aggregates. In contrast, the *APPswe* and *Pgc-1alpha* co-transfected cells presented higher MMP, as revealed by fluorescence localized in small subcellular particles in cellular space (Figure 10F).

To extend the present study findings, we also monitored MMP using flow cytometry. The ratio of aggregates to monomers significantly increased from 1.15 ± 0.17 in cells co-transfected with *APPswe* and *pEnCMV* plasmids to 2.75 ± 0.20 (*p* < 0.001) in cells co-transfected with *APPswe* and *Pgc-1alpha* plasmids. The elevated MMP suggests that PGC-1α has the potential to ameliorate mutAPP induced mitochondrial dysfunction (Figure 10G,H).

### 3.11. Effect of PGC-1α Expression on AD-Like Learning and Memory Abnormalities

- *Days 1~6: Hidden platform at quadrant SE*

**Figure 11:**
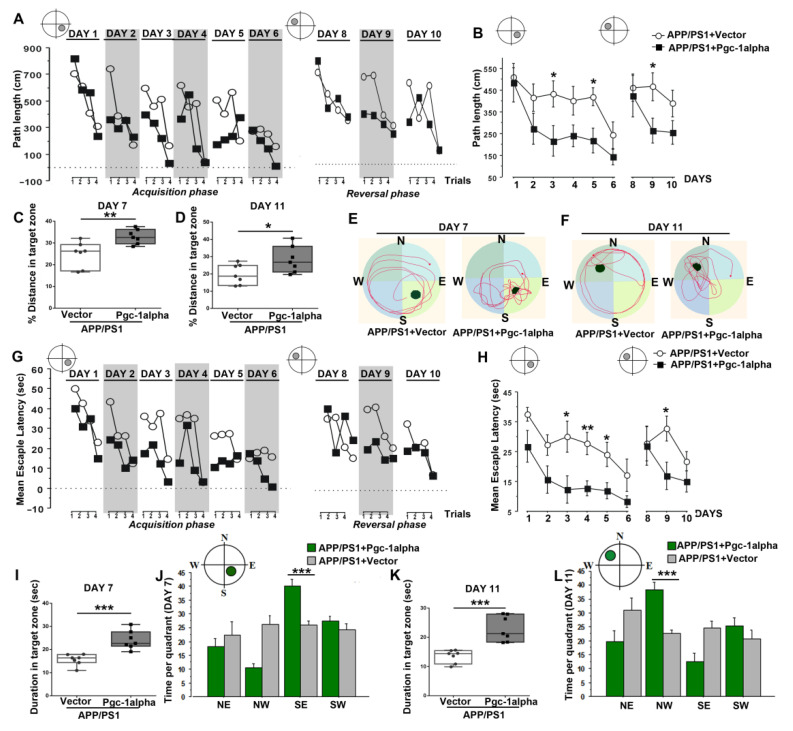
PGC-1α ameliorates spatial learning disability displayed in AD mice. Performance of path length to reach the hidden platform during the acquisition (Day 1–Day 6) and reversal phases (Day 8–Day 10) for animals was expressed as (**B**) a function of days and (**A**) across the 4 trials per day. Distance % in the target zone of the water maze pool during (**C**) probe test 1 (Day 7) and (**D**) probe test 2 (Day 11) are shown in the bar graphs. (**E**,**F**) present the search patterns made by individual animals during probe tests 1 and 2. Performance of escape latency to reach the hidden platform during the acquisition and reversal phases for animals was expressed as (**H**) a function of days and (**G**) across the 4 trials per day. Duration in the target zone of the water maze pool during (**I**) probe test 1 and (**K**) probe test 2 are shown in the bar graphs. Time was recorded in each of the four quadrants to index search preference for the initial platform location during (**J**) probe test 1 and (**L**) probe test 2 for the two treatments. Values were expressed as means ± S.E.M. For each group, *n* = 7–8. Significance levels were set at * *p* < 0.05, ** *p* < 0.01, *** *p* < 0.001 for noted differences between APP/PS1 + Vector and APP/PS1 + *Pgc-1alpha* groups.

During the first six days (Day 1–6) following infusion with AAV-*Pgc-1alpha*, the APP/PS1 animals were trained to acquire the memory of the location of a hidden escape platform fixed to quadrant SE. A 2 × 6 × 4 (treatments × days × trials) ANOVA of path length and escape latency yielded a main effect of treatments [Path length: *F*_(1,10)_ = 6.00, *p* < 0.05; latency: *F*_(1,10)_ = 14.00, *p* < 0.01], of days [Path length: *F*_(5,50)_ = 6.00, *p* < 0.001; latency: *F*_(5,50)_ = 6.00, *p* < 0.001], and of trials [Path length: *F*_(3,30)_ = 11.00, *p* < 0.001; latency: *F*_(3,30)_ = 6.00, *p* < 0.01] (Figure 11A,G).

The effect of AAV-*Pgc-1alpha* infusion on spatial memory during the acquisition phase was most pronounced on Days 3, 4, and 5. Supplementary analysis restricted to each day confirmed that a main effect of treatments emerged on Day 3 [Path length: *F*_(1,13)_ = 5.00, *p* < 0.05; latency: *F*_(1,13)_ = 7.00, *p* < 0.05], Day 4 [latency: *F*_(1,13)_ = 12.00, *p* < 0.01], and Day 5 [Path length: *F*_(1,13)_ = 8.00, *p* < 0.05; latency: *F*_(1,13)_ = 7.00, *p* < 0.05] (Figure 11B,H).

- *Days 7: Probe test 1*

A probe test was conducted on day 7; in this test, the platform was removed, and the animal was placed in the MWM for 60 s. As expected, the *Pgc-1alpha*-treated animals exhibited a strong preference for the SE quadrant, in contrast with the Vector-treated AD animals, during the first probe test (Figure 11E). The search patterns made by individual animals prior to their locating the escape platform was subjected to spatial analysis by calculating the distance and duration spent in the target zone using Student’s T test. As shown in Figure 11C and 11I, there were significant differences in swimming distance (Figure 11C) and latency (Figure 11I) in the target zone between the two treatments (path length: 24.77 ± 2.21 & 32.94 ± 1.26, *p* < 0.01; duration: 15.58 ± 0.90 & 24.08 ± 1.53, *p* < 0.001). Further, we calculated the percent time recorded in each of the four quadrants to index search preference for the initial platform location. Consistently, a 2 × 4 (treatments × quadrants) ANOVA of duration revealed a significant effect of quadrants [*F*_(3,48)_ = 12.00, *p* < 0.001], and of treatments × quadrants [*F*_(3,48)_ = 11.00, *p* < 0.001] (Figure 11J). Supplementary analysis restricted to SE quadrant confirmed that a main treatment effect emerged on APP/PS1 mice [Duration: *F*_(1,12)_ = 23.00, *p* < 0.001] (Figure 11J).

- *Days 8~10: Hidden platform at quadrant NW*

Performance over the subsequent 3 days of testing, in which the escape platform remained hidden but shifted position from SE to NW (Days 8–10), were evaluated. A 2 × 3 (treatments × days) ANOVA of path length and escape latency over this period of training revealed that a significant effect of treatment emerged on Day 9 [Path length: *F*_(1,13)_ = 6.00, *p* < 0.05; Latency: *F*_(1,13)_ = 6.00, *p* < 0.05] (Figure 11B,H).

- *Days 11: Probe test 2*

On day 11, the same probe test was conducted as on day 7. Figure 11D and 11K present the mean distance and duration in the target zone of the treatment mice during probe test 2. As expected, the *Pgc-1alpha*-treated animals exhibited a strong preference for the NW quadrant, in contrast with the Vector-treated AD animals, during the first probe test (Figure 11F). Student’s T test indicated that PGC-1α induced significant increases in distance and duration in the target zone for AD animals [Path length: 19.79 ± 2.21 & 28.33 ± 2.92, *p* < 0.05; duration: 13.46 ± 0.83 & 22.94 ± 1.67, *p* < 0.001]. Further, we calculated the time recorded in each of the four quadrants to index search preference for the initial platform location. A treatments × quadrants ANOVA of time per quadrants yielded a main effect of quadrants [*F*_(3,48)_ = 5.00, *p* < 0.01], and of treatments × quadrants [*F*_(3,48)_ = 9.00, *p* < 0.001] (Figure 11L). Supplementary analysis restricted to NW quadrant confirmed a main effect of treatment emerged on APP/PS1 mice [Duration: *F*_(1,12)_ = 26.00, *p* < 0.001] (Figure 11L).

### 3.12. Effect of PGC-1α Expression on AD-Like Impaired Prepulse Inhibition and Cognition Ability

**Figure 12:**
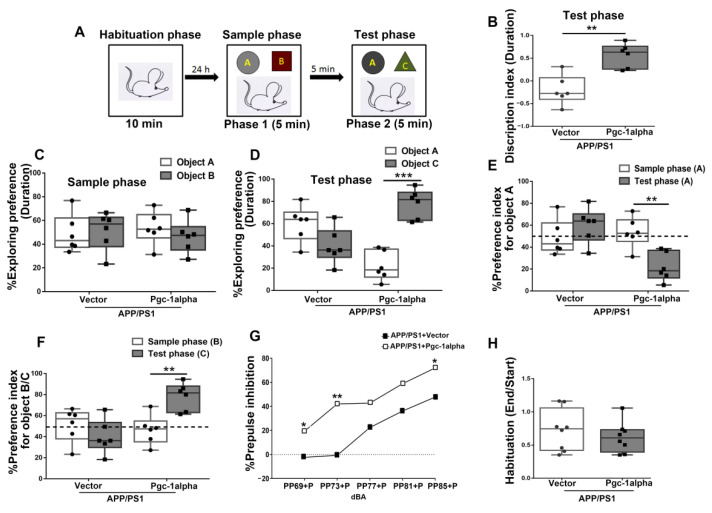
PGC-1α ameliorates novel object learning deficits and sensorimotor gating impairment displayed in AD mice. (**A**) The top panel showed the design of the standard novel object recognition task. During the habituation phase, the animal was allowed to explore an empty arena for 10 min. 24 h later, the animals were exposed to two objects (**A**,**B**) (Sample phase), and then after a 5 min interval, they received a 5 min test in which they were allowed to explore a duplicate of the familiar object (**A**) and a novel object (**C**) (Test phase). (**B**) The time spent exploring the novel object (**C**) for AD mice during the test phase was indicated as Discrimination index (DI). [The test phase: DI = (*D*_C_ - *D*_A_)/(*D*_C_ + *D*_A_)]. (**C**) The treatment (Vector/*Pgc-1alpha*) effect on distinguishing objects A and B for AD mice during the sample phase was indicated by Exploring preference (EP). [The sample phase: EP_A_ = (*D*_A_)/(*D*_A_ + *D*_B_); EP_B_ = (*D*_B_)/(*D*_A_ + *D*_B_)]. (**D**) The treatment (Vector/*Pgc-1alpha*) effect on % EP for AD mice distinguishing object A and C during the test phase was calculated using the formula [The test phase: EP_A_ = (*D*_A_)/(*D*_A_ + *D*_C_); EP_C_ = (*D*_C_)/(*D*_A_ + *D*_C_)]. (**E**) The treatment (Vector/*Pgc-1alpha*) effect on % Preference index (PI) for AD mice recognize the object A during the sample phase was calculated using the formula [The sample phase: PI_A_ = (*D*_A_)/(*D*_A_ + *D*_B_)], while during the test phase, it was calculated using the formula [The test phase: PI_A_ = (*D*_A_)/(*D*_A_ + *D*_C_)]. (**F**) The treatment (Vector/*Pgc-1alpha*) effect on % PI for AD mice recognize the object B during the sample phase was calculated using the formula [The sample phase: PI_B_ = (*D*_B_)/(*D*_A_ + *D*_B_)], while recognizing object C during the test phase was calculated using the formula [The test phase: PI_C_ = (*D*_C_)/(*D*_A_ + *D*_C_)]. (**H**) The mean startle magnitudes obtained in the first six and last six pulse-alone (120 dB_A_) trials are shown in the bar graph. (**G**) % PPI was expressed as a function of prepulse intensity (69, 73, 77, 81, and 85 dB_A_) for mice of the two treatments. % PPI = [(pulse alone-(prepulse plus pulse))/pulse alone × 100%]. Values were expressed as means ± S. E. M. For each group, *n* = 7–8. Significance levels were set at * *p* < 0.05, ** *p* < 0.01, *** *p* < 0.001 for noted differences between two treatments/objects/phases.

To confirm the role of AAV-*Pgc-1alpha* infusion in AD-like behavioral abnormalities, the NOL test was applied to evaluate the cognition ability. We first evaluated description index (DI) based on duration calculated using the formula [(time at object C-time at object A)/(time at object C + time at object A) during the test phase. The unpaired Student’s *t*-test indicated a significant increase in DI of *Pgc-1alpha* in contrast with the Vector-treated AD mice [-0.20 ± 0.13 & 0.56 ± 0.11, *p* < 0.01] (Figure 12B).

We also analyzed the treatment (Vector/*Pgc-1alpha*) effect on % exploration preference (EP) for object A or B based on duration calculated using the formula [(time at object A/B)/(time at object A + time at object B) × 100% in APP/PS1 mice during the sample phase (Figure 12C). The results were supported by a two-way (treatments × object) ANOVA, which yielded neither a treatment (*p* = 1.00) nor an object (*p* = 0.72) effect.

Consistently, we further analyzed the treatment (Vector/*Pgc-1alpha*) effect on % EP for object A or C based on duration in APP/PS1 mice during the test phase (Figure 12D). Specifically, % EP for object A was calculated using the formula [time at object A/(time at object A + time at object C) × 100% and for object C was calculated using the formula [time at object C/(time at object A + time at object C) × 100% during the test phase. The results were supported by a two-way (treatments × objects) ANOVA, which yielded a main effect of object [*F*_(1,20)_ = 9.00, *p* < 0.01], as well as an interaction of treatments × objects [*F*_(1,20)_ = 40.84, *p* < 0.001]. The unpaired Student’s *t*-test indicated a significant difference in the exploration duration between the familiar object A and the novel object C in *Pgc-1alpha*-treated mice [21.91 ± 5.34 & 78.09 ± 5.34, *p* < 0.001].

Figure 12E shows the treatment (Vector/*Pgc-1alpha*) effect on % Preference index (PI) for object A, calculated using the formula [time at object A/(time at object A + time at object B/C) × 100% between the two phases in APP/PS1 mice. *Pgc-1alpha*-treated mice paid less attention to object A when test phase (phase 2) was compared with sample phase (phase 1) [53.56 ± 5.65 & 21.91 ± 5.34, *p* < 0.01]. These results were supported by a two-way (treatments × phases) ANOVA, which yielded a main effect of treatment [*F*_(1,20)_ = 7.60, *p* < 0.05], as well as an interaction of treatments × phases [*F*_(1,20)_ = 12.70, *p* < 0.01].

Figure 12F presents the treatment (Vector/*Pgc-1alpha*) effect on % PI for object B during phase 1 and for object C during phase 2 in APP/PS1 mice. *Pgc-1alpha*-treated mice paid more attention to object C during phase 2 than object B during phase 1 [39.86 ± 6.56 & 78.09 ± 5.34, *p* < 0.01], indicating that the *Pgc-1alpha* treatment ameliorated novel object learning ability of APP/PS1 mice. These results were supported by a two-way (treatment × phase) ANOVA, which yielded a main effect of treatment [*F*_(1,20)_ = 7.60, *p* < 0.05], as well as an interaction of treatments × phases [*F*_(1,20)_ = 12.70, *p* < 0.01].

One-way ANOVA of the mean habituation, calculated using the formula [(Last 6 pulses (End))/First 6 pulses (Start)], showed no significant difference between the two treatments during the startle habituation test (Figure 12H).

A 2 × 5 (treatments × Prepulse intensity) mixed ANOVA yielded a main effect of treatment in the PPI test [*F*_(1,12)_ = 7.06, *p* < 0.05]. As revealed in Figure 12G, PGC-1α expression ameliorated PPI in an intensity-dependent manner. Specifically, a prominent elevation in percent PPI with prepulse stimuli of intensities 69, 73 and 85, corresponding to 4, 8 and 20 dBA above background was inducible by infusion with AAV-*Pgc-1alpha* than AAV-*Vector* in AD mice (Figure 12G).

## 4. Discussion

The complexities that underlie the neuronal degeneration and cognitive impairment of Alzheimer’s disease have yet to be completely understood, although many factors in disease pathologies have been identified. There is growing body of evidence that mitochondria are potentially a foremost event for energy failure and for subsequent axonal degeneration in AD [25].

In this study, using TEM technology, we showed that mutAPP induced mitochondrial swelling and damage in neurons. Based on the fact that mitochondrial morphology is tightly controlled by the balance of mitochondrial fission and fusion [1], AD-associated changes in mitochondrial morphology should be caused by the imbalanced expression of fusion and fission proteins. Previous findings have demonstrated the association of amyloid-beta overproduction with the changes of mitochondrial fission/fusion proteins in hippocampus, one of the AD-specific brain regions [1]. Here, in AD cortical neurons, supplementary evidence was provided that mutAPP-induced dynamic balance between the fission and fusion of mitochondria is greatly shifted toward the fissive stage. Further, the changes in mitochondrial dynamics in AD are revealed as a reduction in the expression of fusion protein and an increase in the levels of fission protein.

Being one of the dynamic organelles, the balance between mitochondrial fission and fusion not only controls mitochondrial morphology but also regulates its distribution [1]. An important finding by Xinglong Wang et al. was that manipulations of mitochondrial fission and fusion proteins in neuronal cells to mimic their expression changes in AD could cause a similar abnormal mitochondrial distribution pattern to that caused by oligomeric amyloid-β-derived diffusible ligands [1]. Thereafter, we examined mitochondrial distribution patterns in AD cells. Consistently, our studies confirmed that mitochondrial redistribution from an evenly distributed pattern to perinuclear accumulation occurs in AD neurons. The abnormal mitochondrial distribution in axons was accompanied by reduced protein expression of MNF2, KIF5A and KIF5B, which are responsible for mitochondrial transport, adaptor binding, and axonal maintenance [21].

Given that mitochondria are normally abundantly localized at synaptic terminals, presumably reflecting the intense ATP demands of an active neuron engaged in synaptic transmission and need of calcium buffering at these sites, perturbations in mitochondrial distribution are correlated with synaptic loss in neurodegenerative diseases [1]. Here, we confirmed a remarkable increase in neuronal apoptosis in AD animals and cells. It has been shown that mitochondrial swelling is one of the hallmarks of multiple forms of necrotic cell death [26]. Mendoza reported that matrix calcium (Ca^2+^) triggered the opening of mitochondrial permeability transition pores, which was followed by MMP dissipation, and then the mitochondria began to swell, rendering them dysfunctional [26]. Intersections between mitochondrial swelling and mitochondrial fusion/fission have been studied independently in the past, although recently, a link between the outer membrane pore and fusion events was proposed, i.e., two major classes of structural changes in mitochondria are correlated with apoptosis [27]. As expected, the neuronal apoptosis is followed by mitochondrial swelling and lowered MMP, suggesting that mutAPP-induced dynamic imbalance and abnormal distribution of mitochondria are unable to meet the metabolic demands of the cells.

Impaired mitochondrial transport and mitochondrial dysfunction are prominent features of AD, reflecting the profound influences of brain mitochondria in the pathogenesis of AD. These afford a new perspective on potential attractive therapeutics for the disease that could rescue the AD-relevant dynamic abnormalities and impaired mitochondrial transport that precede axonal degeneration. PGC-1α has been coined the “master regulator of metabolism” based on its ability to induce gene programs controlling mitochondrial biogenesis and antioxidant production [15]. Emerging evidence indicates that it is strongly expressed in tissues with a high energy demand, including brown adipose tissue, skeletal muscle and brain [28]. The role of PGC-1α in neurodegenerative disease is an emerging field; the function of PGC-1α in AD has been suggested but not studied in depth [29]. Our previous study revealed that PGC-1α contributes to the improvement of AD pathophysiology by upregulating VDR expression and ameliorating DNA oxidative damage [10]. Thus far, the role of PGC-1α in AD-relevant neurobehavioral dysfunction and its potential value in restoration of mitochondrial dynamics remain largely unknown.

### 4.1. PGC-1α Ameliorates AD-Like Neurobehavioral Abnormalities and Inhibits Neuronal Apoptosis

To investigate the neuroprotective function of PGC-1α in AD and its potential mechanisms, we forced PGC-1α overexpression in the LPtA cortex of 2×Tg-AD mice by microinjecting pAAV-MCS-*Ppargc1α*-m-FLAG-HA vector. LPtA is responsible for integrating sensory input to form a single perception (cognition) on the one hand while also forming a spatial coordinate system to represent our world on the other hand [30]. There is a range of clinical manifestations following injury to LPtA, such as an inability to understand spatial relations as well as impaired attention, working memory and social cognition [30,31]. As such, it was selected as the research region in the present study.

Using the 2×Tg-AD mouse line, combined with AAV-*Ppargc1α* microinjection, we successfully forced PGC-1α overexpression in the LPtA of AD brain, as indicated by the strong expression of HA-labeled PGC-1α. Thereafter, we explored the impact of PGC-1α on AD-relevant neurobehavioral manifestations. PPI is a neurological phenomenon in which a weak initial stimulus reduces the level of responses to a subsequent stronger stimulus [15]. It acts as a reliable tool for predicting working memory performance in mice [32]. The NOR task is very useful for studying short-term memory [33]. By analyzing the behavioral results from PPI and NOL, we confirmed that PGC-1α activation ameliorated AD-related sensorimotor gating impairment and working memory deficits. The behavioral studies from MWM have long suggested evaluating spatial learning ability [34]. In the present study, we observed that PGC-1α activation prominently reversed learning and memory abnormalities displayed by 2×Tg-AD mice.

Given the evidence that learning and memory deficits are caused at least in part by neuronal degeneration and loss [14], the study of apoptosis-related proteins is necessary for evaluating the neuroprotective function of PGC-1α. Several studies have indicated that the Bcl-2 protein is protective against apoptosis, whereas the BAX protein is believed to induce apoptosis [35]. Therefore, we assessed the expressions of Bcl-2 and BAX and found that PGC-1α affected AD-related apoptosis, as revealed by a lowered apoptosis index (BAX/Bcl-2), a reduced apoptosis rate and an amelioration of neuronal damage in AD. As we all know, a large amount of energy is necessary for neurons to satisfy their normal function and activity. In order to meet this demand, mitochondrial fission, fusion and movement events (mitochondrial dynamics) control mitochondrial morphology and distribution [1]. Along this line of reasoning, we suggest that the mechanisms of PGC-1α ameliorate AD-relevant neuronal apoptosis and neurobehavioral abnormalities are likely through regulating mitochondrial dynamics.

### 4.2. PGC-1α Rescues Impaired Mitochondrial Dynamics and Mitochondrial Dysfunction in AD

A large number of studies indicate that the balance of fission and fusion in AD is greatly shifted toward fission and that as a result, affected neurons contain abnormal mitochondria that are unable to meet the metabolic demands of the cells [23], the roles of PGC-1α in AD and its involvement in the regulation of dynamical balance is largely unknown.

Recent studies demonstrated that activating SIRT1-PGC-1α pathway prevented mitochondrial fission in diabetic hearts by negatively regulating the expression of Drp1 [36]. Notably, Soriano et al. reported that PGC-1α is involved in the mitochondrial regulation pathway, likely through stimulating the activity of MFN2 promoter [12]. In light of that, we suggest that PGC-1α activation can be a feasible balancing strategy for mitochondrial fission and fusion. Using immunoblot technologies, we observed that PGC-1α activation increased the levels of OPA1, MFN1 and MFN2 but reduced the expression of DRP1 and FIS1. Consistently, AAV-*Pgc-1alpha* infusion led to an obvious inhibition in the fissive stage of mitochondria in cortical neurons of 2×Tg-AD mice. That means that PGC-1α rescued AD-relevant mitochondrial imbalance between fission and fusion both in vitro and in vivo.

Mitochondrial dynamics also impact mitochondrial distribution, since both fission mutants (i.e., Dlp1) with elongated mitochondria and fusion mutants (i.e., OPA1) with short, rounded mitochondria can cause mitochondrial distribution changes [1]. In an attempt to address the potential role of PGC-1α on mitochondrial distribution, MitoTracker was used to label mitochondria, it was observed that mutAPP-induced mitochondrial depletion from remote cytoplasmic areas and axons; however, when transfected with *Pgc-1alpha* plasmids, cells were observed in which mitochondria were distributed evenly throughout the cytoplasm and axons. *Mfn-2* gene knockout or *Mfn-2* disease-expressing mutants affect the transport of axonal mitochondria [22], and on this basis, PGC-1α possibly contributes to the rescue underlying the defection of axonal integrity and reduction in synaptic function in AD [22] by stimulating the activity of the *Mfn-2* promoter [12].

To further explore the detailed regulatory mechanisms of PGC-1α in mitochondrial transport, MFN2, KIF5A and KIF5B, which are key molecular machinery in facilitating anterograde axonal mitochondrial transport, were examined; we showed that PGC-1α expression downregulated MFN2, KIF5A and KIF5B levels and that the restoration of MFN2, KIF5A and KIF5B corrected mutAPP-induced impairments in axonal mitochondrial transport. Previous findings have reported that *Mfn-2* deficiency impairs mitochondrial transport and downregulates motor protein, including KIF5A and KIF5B, expression in human spinal motor neurons [13]; this combined with the role of PGC-1a activating *Mfn-2* promoter suggested that the restoration of impaired mitochondrial transport by PGC-1α is mediated, at least partially, by MFN2-regulated alteration of motor protein.

In has been reported that mitochondrial dysfunction is pivotal for axonal degeneration in many neurodegenerative diseases [37]. Because mitochondrial function is regulated by the dynamics of its membrane fusion-fission, imbalanced dynamics of mitochondria potentially is a foremost event for energy failure and for subsequent axonal degeneration in AD [6,25]. It is not entirely clear, though, how mitochondrial fission causes mitochondrial dysfunction. One possible explanation was that excessive mitochondrial fission damaged the structure and the integrity of the mitochondrial membrane and caused mitochondrial dysfunction [38]. Excessive mitochondrial fission may cause changes to the curvature of the cristae membrane that could impact the assembly of electron transport chain complexes and supercomplexes and affect their stability and activity [38]. Moreover, mitochondrial distribution in AD cells is perinuclear, with few mitochondria in the distal processes, where they are needed for exocytosis, ion channel pumps and synaptic function, among other things. Hence, we suggest that the forced expression of PGC-1α not only ameliorates dynamical abnormalities of mitochondria but also benefits mitochondrial morphology and function. As expected, a significantly higher percentage of swollen mitochondria with broken membranes was observed in the cortical neuron of 2×Tg-AD mice, which was correlated with reduced MMP. However, when PGC-1α overexpressed, an obvious amelioration of mitochondrial swelling was evidenced on electron microscopy. Importantly, PGC-1α expression further rescued mitochondrial functional deficit in AD cells, as revealed by the restoration of mitochondrial MMP in vitro. These results reflect that the mechanisms of PGC-1α inhibiting AD-related apoptosis and neurobehavioral abnormalities are likely through the improvement of mitochondrial dynamics, transport and dysfunction.

## 5. Conclusions

Taken together, these findings support the notion that abnormal mitochondrial dynamics plays a causal role in mitochondrial dysfunction and AD-related pathological and cognitive impairments and demonstrate PGC-1α as a potential therapeutic target for future drug development for AD. The intervention strategy of PGC-1α in AD is likely through rebalancing the mitochondrial fission and fusion, as well as rescuing the abnormal mitochondrial distribution pattern and function.

## Figures and Tables

**Table 1:** Summary of clinicopathological characteristics of human cases.

Group	*n*	Age (yrs)	Gender (M/F)	Duration (yrs)	Education (yrs)	DRS (Mean)	MMSE (Mean)
Control	3	87.0 ± 4.4	1/2	0	11.2 ± 2.6	141.4 ± 4.33	29.21 ± 0.6
AD	4	85.0 ± 3.8	2/2	9.8 ± 1.6	12.3 ± 1.3	39.67 ± 10.13	7.4 ± 2.3

Note: yrs, years; DRS, dementia-rating scale evaluation; MMSE, Mini Mental State Examination evaluation.

## Data Availability

The data that support the findings of this study are available from the corresponding author upon reasonable request.
